# Assessing the perceived influence of religion on brain health among adults in the United Arab Emirates—the Global Brain Health Survey: a cross-sectional study

**DOI:** 10.3389/fpsyg.2025.1526367

**Published:** 2025-04-02

**Authors:** Iffat Elbarazi, Aminu S. Abdullahi, Karim Abdel Aziz, Emmanuel Stip, Isabelle Budin-Ljøsne, Javaid Nauman

**Affiliations:** ^1^Institute of Public Health, College of Medicine and Health Sciences, United Arab Emirates University, Al-Ain, United Arab Emirates; ^2^Department of Psychiatry, College of Medicine and Health Sciences, United Arab Emirates University, Al-Ain, United Arab Emirates; ^3^Department of Psychiatry, University of Montréal, Montreal, QC, Canada; ^4^Institut Universitaire en Santé Mentale de Montréal Université de Montréal, Montreal, QC, Canada; ^5^Department of Food Safety, Division of Climate and Environmental Health, Norwegian Institute of Public Health, Oslo, Norway; ^6^Department of Circulation and Medical Imaging, Faculty of Medicine and Health Sciences, Norwegian University of Science and Technology, Trondheim, Norway; ^7^Healthy Living for Pandemic Event Protection (HL-PIVOT) Network, Chicago, IL, United states

**Keywords:** brain health, religion, religiosity, GBHS, UAE

## Abstract

**Background:**

A healthy brain is essential for independent and participatory life. Religion may play a key role in brain health. This study investigated the influence of religion on brain health among adults in the United Arab Emirates (UAE).

**Methods:**

This was a cross-sectional study among adults in the UAE based on the Global Brain Health Survey (GBHS). Information on demographics, knowledge and beliefs about brain health, and religious perceptions and practices related to brain health was collected. Data were summarized using frequencies and percentages. Logistic regression analysis was used to identify factors associated with religious activities and attitudes toward brain health, and results are presented as adjusted odds ratios (OR) and 95% confidence intervals (CI).

**Results:**

A total of 887 participants (65% women) were included. About 78% of women and 73% of men believed that religion strongly influences brain health. About 47% of participants reported frequent practice of religion for their brain health. Frequent thoughts about one’s brain health (OR = 2.52, 95% CI = 1.47–4.31), frequent engagements in religious activities (OR = 33.42, 95% CI = 18.58–60.11), being married (OR = 0.46, 95% CI = 0.23–0.90), and having had COVID-19 (OR = 0.51, 95% CI = 0.27–0.97) were associated with purposeful use of religious activities for brain health.

**Conclusion:**

Our study found a significant link between religious practices and brain health, suggesting that faith- and spirituality-based approaches may be important for addressing brain health issues. These findings highlight the need for programs that incorporate religious beliefs to improve brain health, offering practical solutions for communities and healthcare providers.

## Introduction

Individuals’ independence and ability to engage in important matters as well as their participation in various aspects of life is intrinsically linked to the healthy functioning of their brain (Alchalabi and Prather, 2021). Despite being essential to human functioning and overall health, brain health has no consensus definition and varies across organizations (Alchalabi and Prather, 2021). For instance, the World Health Organization (WHO) defines brain health as “the state of brain functioning across cognitive, sensory, social–emotional, behavioral and motor domains, allowing a person to realize their full potential over the life course, irrespective of the presence or absence of disorders” (World Health Organization, 2022). Similarly, the Center for Disease Control and Prevention described a healthy brain as one capable of performing all cognitive processes, including learning, intuition, judgement, language, and memory (Centers for Disease Control and Prevention, 2009). The American Heart Association emphasizes age-relative performance, defining brain health as “average performance levels among all people at that age who are free of known brain or other organ system diseases” (Gorelick et al., 2017). In a recent effort to harmonize these perspectives, Chen et al. defined brain health as “a life-long dynamic state of cognitive, emotional and motor domains underpinned by physiological processes” (Chen et al., 2022). These varying definitions reflect the multidimensional nature of brain health, and its importance for overall well-being.

The effects of poor brain health are evident in the high prevalence of neurological disorders, such as stroke, meningitis, dementia, and migraines, which currently account for the highest proportion of disability, and the second highest cause of death globally (Feigin et al., 2020). With population aging and other demographic shifts, this burden is expected to increase, and it is estimated that almost one-third of the world’s population will experience some form of neurological disorder during their lifetime (Feigin et al., 2020). Despite the significance of a healthy brain and the detrimental consequences of an unhealthy one, brain health has not received the attention it deserves, and policy responses to address brain health issues are insufficient (Kolappa et al., 2022; Gorelick et al., 2024).

Brain health is influenced by a range of factors including but not limited to social determinants of health such as education, employment status and quality of work, maternal and child health, and healthy aging (Allen et al., 2014). People’s perceptions of brain health are different and may be influenced by different factors and are highly associated with mental health as well as social and cultural practices (Budin-Ljøsne et al., 2022). Furthermore, engagement in religious activities can have a positive impact on brain health (Moritz et al., 2006; Gonçalves et al., 2015; Koenig, 2009). For instance, regular religious attendance has been found to reduce depressive symptoms in men and women aged 50 years and above (Orr et al., 2019). Similarly, a study among the elderly in Malaysia showed that religiosity, measured in terms of routine engagements in various religious activities, was associated with better cognitive function and overall quality of life (Amir et al., 2022). Additionally, religiosity has been identified as an important aspect of quality of life in the elderly, allowing them to accept physiological declines, including those related to brain health, and to adapt to negative changes while being contented and satisfied with unfavorable changes (van Leeuwen et al., 2019).

Despite these findings, the relationship between religion and brain health in the adult population has not been extensively studied, and there is a lack of a clear understanding regarding this association in culturally and religiously diverse settings like the United Arab Emirates (UAE). The UAE’s unique demographic, cultural and religious context, where approximately 11% of the population are citizens, about two-thirds are Muslims, and the remainder adhere to diverse religious beliefs such as Christianity, Hinduism, Buddhism and others (United States Department of State, Office of International Religious Freedom, 2022), provides an appropriate setting to examine the association between religion and brain health. However, limited research has been conducted on how religious practices may influence the brain health in the UAE population. This gap is significant, as understanding the role of religion in brain health could inform culturally tailored interventions and policies to promote cognitive well-being in the country and beyond in similar settings. Therefore, we aimed to investigate the influence of religion on brain health among the adult population in the UAE, and identify factors associated with the use of religious practices to maintain and enhance brain health.

## Materials and methods

We conducted an online cross-sectional study in the UAE based on the updated version of the Global Brain Health Survey (GBHS) by the Lifebrain consortium (Budin-Ljøsne et al., 2020). The GBHS was conducted in Europe during the year 2020 to explore people’s interest in brain health, and to investigate what people purposefully do to improve or maintain their brain health, how motivated they are to take tests to learn about their brain health, which activities they are willing (or not willing) to engage in, and what kind of support they would need to make lifestyle changes. The survey respondents are also asked to select public health measures they see as most efficient to promote brain health. The study has collected more than 27,000 responses across Europe and the data have been analyzed by the Lifebrain team (Budin-Ljøsne et al., 2022; Carver et al., 2022). A detailed account of the GBHS is provided elsewhere (Budin-Ljøsne et al., 2020).

The GBHS questionnaire starts with an information page defining brain health and detailing the purpose of the survey and the main desired outcomes, and includes questions related to demographics, knowledge and beliefs about brain health, attitudes toward brain health and lifestyle practices followed or believed to improve brain health. In the updated version of GBHS used in the present study, additional questions related to religious and cultural practices were added due to the overall social, religious, and cultural diversity and being a majority Muslim country. After incorporating the feedback from the Lifebrain consortium related to the additional questions, we translated the questionnaire into Arabic where back-to-back translation was performed by two native Arabic speakers. We have followed the scale translation process as detailed by Koenig and Al Zaben (2021) which included the preparation, then the forward translation, the back translation and the reconciliation by the research team who spoke English and Arabic languages fluently and who agreed on the final copy. After translation, the tool was piloted with experts from the research team network and community members to assess language clarity and comprehension. Necessary adjustments were made, and the Lifebrain team reviewed and approved the final updated GBHS. It is worth noting that the tool was not constructed originally to measure religion and spirituality impact but after adaptation, we have introduced the questions that are related to religion and health being highly impactful on people’s behaviour and beliefs.

We used a convenience sampling approach to ensure broad accessibility and participation which helped to include a diverse range of respondents from across the UAE. The study’s inclusion criteria encompassed adults of any nationality, and aged 18 years and above. One Arabic and one English version of the questionnaire were uploaded on the SurveyMonkey platform, and data were collected between September 2021 and January 2022. To reach potential participants, the research team employed various strategies including utilization of social media platforms and their personal connections to distribute the survey. A QR code was generated to facilitate easy access to the questionnaire. Additionally, a flyer containing study details and the QR code was designed and shared across university campuses, shopping malls, coffee shops, and workplaces, and on various social media platforms such as Instagram, Facebook, Twitter, LinkedIn, and Reddit. The questionnaire was approved by the Lifebrain consortium, and ethical approval of the study was obtained from the UAE University Social Science Ethics Committee (ERS_2020_7231), and the study was strictly conducted in accordance with the relevant guidelines and regulations.

The survey was initially available for a duration of 6 weeks; however, to enhance the response rate, the timeframe was extended to a further 3 months. We actively engaged students and research assistants to aid in the dissemination of the survey and urged them and the team members to share the survey through their social media platforms. To further incentivize participation, an Instagram competition was introduced, offering the chance to win gift vouchers. The winners of these vouchers were selected through a raffle process involving participants who had taken part in the survey.

Due to the social, religious, and cultural diversity of the UAE, questions related to religious and cultural practices were added to the updated version of the GBHS questionnaire. For example, participants were asked the following questions related to religion and were provided with the relevant options to choose from:

1. “In your opinion, to what extent does religion have an influence on brain health?” Options: “very strong influence,” “strong influence,” “moderate influence,” “weak influence,” and “no influence”.
2. “How often do you engage in religious activities (prayers, gatherings, fasting)?” Options: “frequently,” “occasionally,” “rarely,” and “never”.
3. “Do you do religious activities (pray, script reading, go to sacred places) purposefully for your brain health?” Options: “frequently,” “occasionally,” “rarely,” and “never”.
4. “Imagine you undergo a brain health test, and it shows that you have a risk of developing a brain disease, would seeking a religious person/healer’s help be your most likely reaction?” Options: “Definitely yes,” “fairly likely,” “fairly unlikely,” “definitely no”.
5. “Your doctor says that you can reduce your risk of developing a brain disease by changing your lifestyle. How likely would you do more cultural/religious activities?” Options: “very likely,” “somewhat likely,” “I already do that,” “somewhat unlikely,” “very unlikely,” “not applicable”.
6. “Imagine you decide to change your lifestyle to maintain or improve your brain health, what kind of assistance would you need?” with one of the choices being “support from a religious organization”.
7. “Do you perform any religious activities to preserve your brain health?” with a list of the activities “Pray,” “go to mosque/church/temple/other,” “read Quran/Bible/Old Testament,” “fast,” “memorize Quran/Bible/Old Testament,” “go to religious classes,” “go to Pilgrimage/Umrah,” “go to Sheikh/Priest for ruqya/yoga” and the options “extensive,” “moderate,” “not at all” to indicate the extent.
8. “What are the measures that you have taken to maintain good brain health during the COVID-19 pandemic?” with “religious activities (prayer, script reading, going to worship places)” as one of the measures. Options: “frequently,” “occasionally,” “rarely,” and “never” to assess the frequency.

Data were summarized and presented as frequencies and percentages. We used multivariable logistic regression analysis to identify factors associated with religious activities and those associated with the likelihood of seeking religious help upon a brain health test that indicates a risk of developing a brain disease. Variables with a *p < 0.25* in the univariate analysis were included in the final multivariable models. Data were presented as adjusted odds ratios (aOR) and associated 95% confidence intervals (CI). Statistical Package for Social Sciences (SPSS, version 28.0) was used for the analysis.

## Results

### Demographics

A total of 887 participants were included in the analyses of which the majority were women (65%) (Table 1). The highest representation was among those between the ages of 18 and 25 years, comprising 45.5% of the participants. Nearly 73% of the participants were residing in Abu Dhabi Emirates. About 41 and 20% of participants had a university or college degree and a postgraduate degree, respectively. Slightly above half (52%) were single and academicians made up most of the participants with about 37%.

**Table 1:** Demographic characteristics of the participants (*N* = 887).

Characteristics	Frequency	Percentage
Age		
18–25	404	45.5
26–40	240	27.1
41–60	203	22.9
>60	40	4.5
Gender		
Male	310	35.3
Female	568	64.7
Emirate		
Abu Dhabi	633	72.6
Dubai	108	12.4
Ajman	31	3.6
Sharjah	43	4.9
Ras Al Khaimah	14	1.6
Fujairah	21	2.4
Um Al Quwain	22	2.5
Education		
Lower than university/college	334	38.3
University/college degree	361	41.4
Postgraduate	177	20.3
Marital status		
Single	453	52.4
Stable relationship, not married	36	4.2
Married	357	41.3
Divorced/separated	10	1.2
Widow/widower	9	1.0
Main occupation		
Teacher	121	13.9
Healthcare professional	128	14.7
Academics	319	36.6
Researcher	80	9.2
White collar	169	19.4
Blue collar	55	6.3

White collar (office workers, engineers, lawyers, accountants, etc.); Blue collar (labourer, driver, factory worker, cleaner, etc.).

### Religious engagement

**Figure 1:**
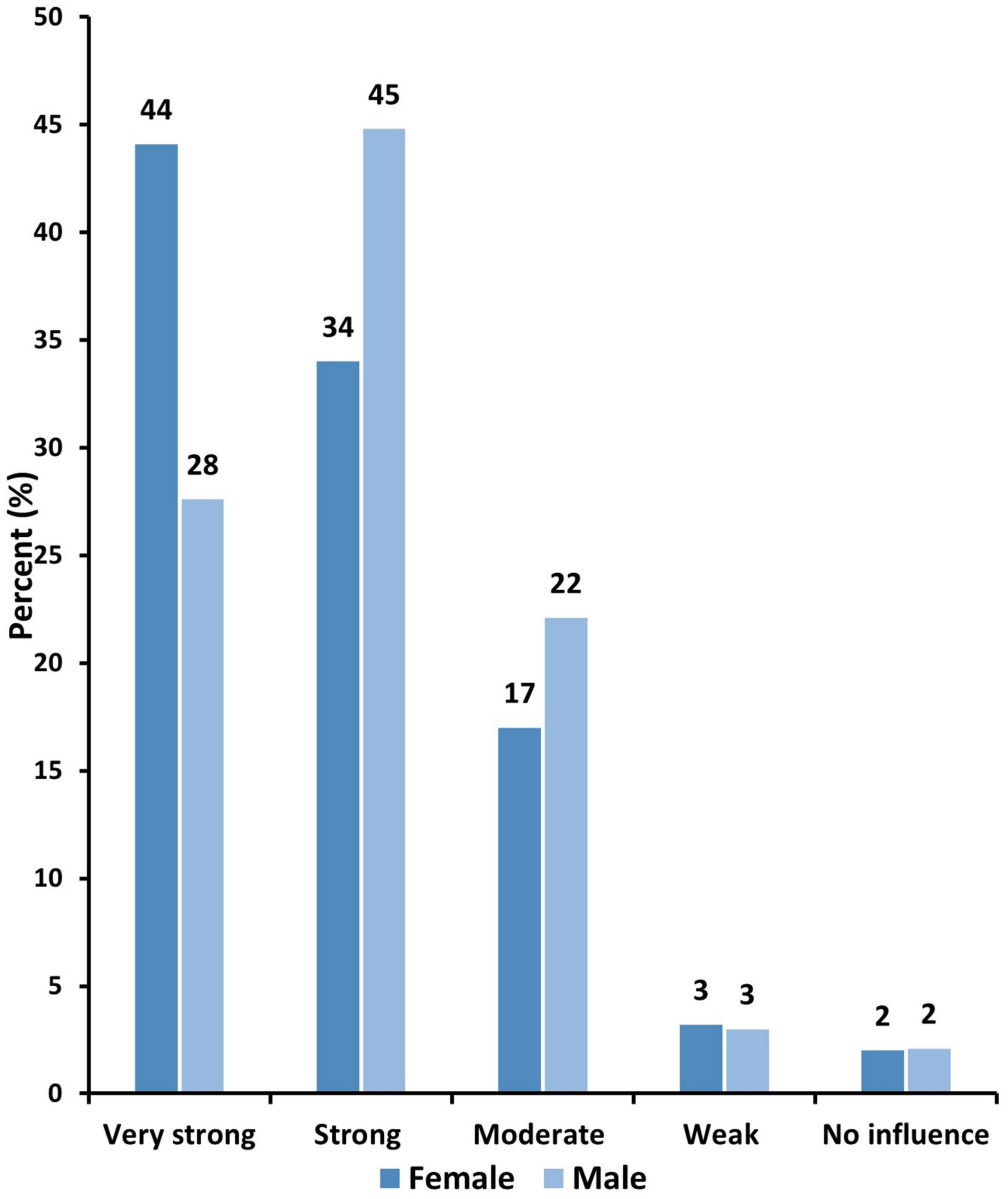
Perceived religious influence on brain health by gender (*N* = 878).

**Figure 2:**
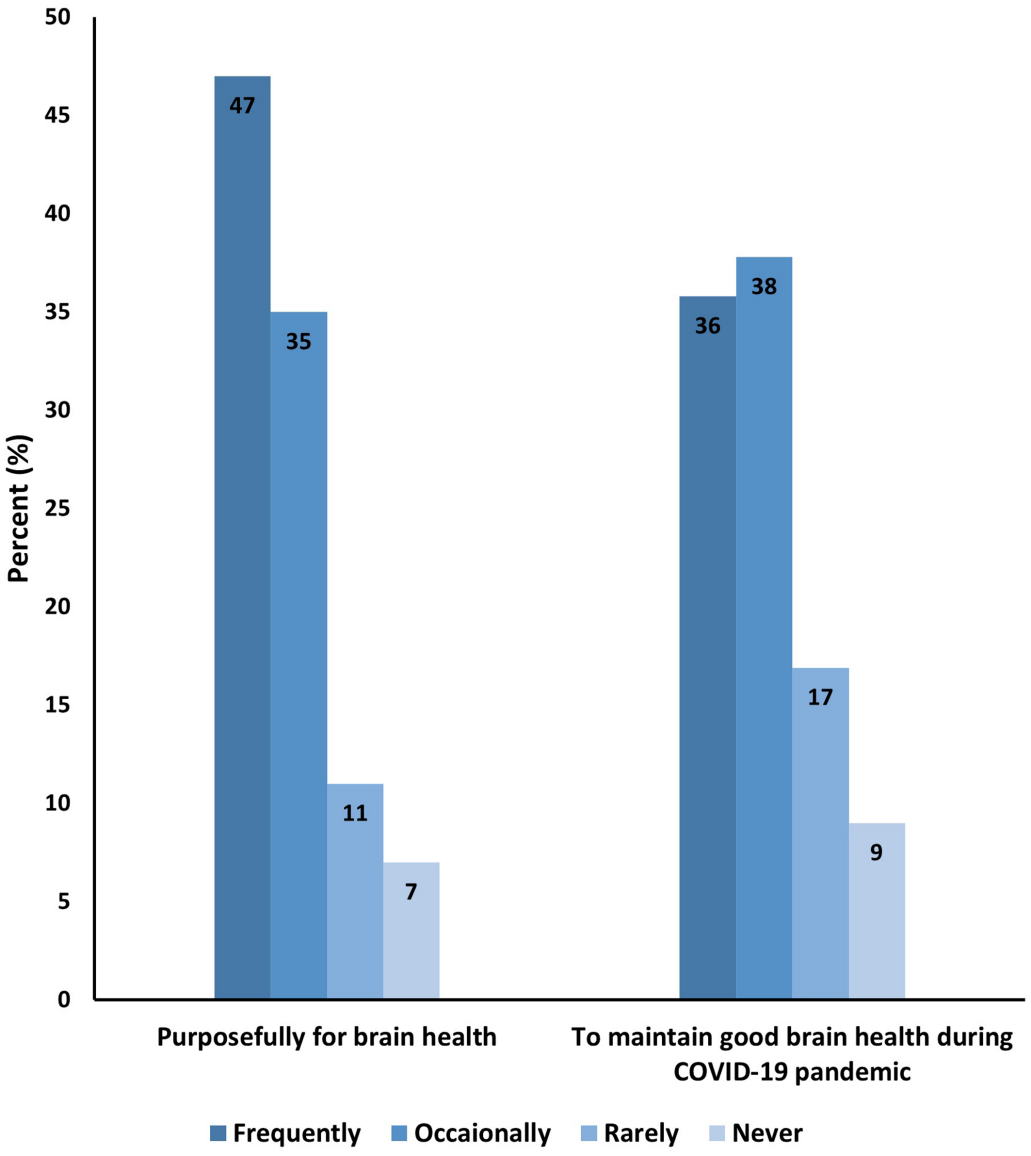
Practice of religious activities to improve and maintain brain health (*N* = 887).

**Figure 3:**
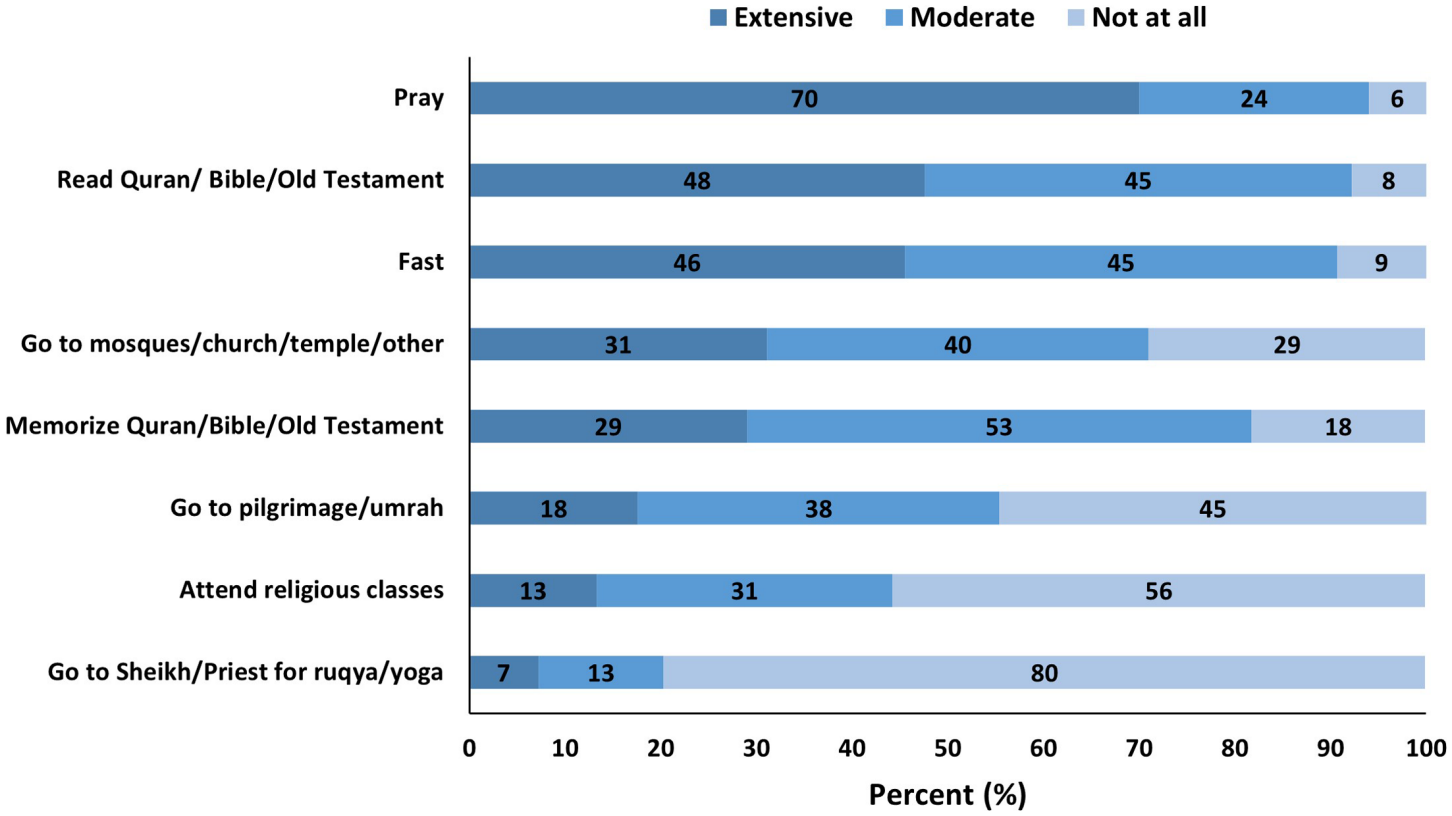
Religious practices for brain health preservation.

About 78% of women and 73% of men believed that religion has a very strong or strong influence on brain health (Figure 1). Almost half of the participants (47%) reported frequent practice of religion purposefully for their brain health (Figure 2). Overall, prayers were the most common form of religious activity performed extensively by the participants to preserve their brain health as reported by 70% of the participants. This is followed by reading holy books such as Qur’an and Bible (48%), fasting (46%), attending worship places such as mosques, churches, and temples (31%), memorizing holy books such as Qur’an and Bible (29%), going on religious pilgrimage (18%), attending religious classes (13%), and lastly going to Sheikh or Priest for Ruqya (religious healing) or yoga (7%) (Figure 3).

### Factors associated with religious practices for brain health

Participants who reported thinking frequently about their brain health (aOR = 2.52, 95% CI = 1.47–4.31), and those who reported frequently engaging in religious activities (aOR = 33.42, 95% CI = 18.58–60.11) were more likely to practice religion purposefully for brain health. However, married people (aOR = 0.46, 95% CI = 0.23–0.90) and those who ever had COVID-19 (aOR = 0.51, 95% CI = 0.27–0.97) were less likely to engage in the purposeful use of religious activities for brain health (Table 2).

**Table 2:** Factors associated with the frequent purposeful use of religious activities for brain health.

Predictors	OR^a^ (95% CI)	OR^b^ (95% CI)
Age		
18–40	Ref.	Ref.
>40	0.61 (0.45–0.82)	1.13 (0.55–2.33)
Gender		
Male	Ref.	Ref.
Female	1.90 (1.43–2.52)	0.73 (0.40–1.34)
Highest education		
Diploma or lower	Ref.	Ref.
University/College	1.73 (1.28–2.33)	1.52 (0.83–2.81)
Postgraduate	1.16 (0.80–1.68)	0.94 (0.47–1.88)
Marital status		
Unmarried	Ref.	Ref.
Married	0.59 (0.44–0.77)	**0.46 (0.23–0.90)**
Had COVID-19		
No	Ref.	Ref.
Yes	0.55 (0.40–0.77)	**0.51 (0.27–0.97)**
Think about brain health		
Infrequently	Ref.	Ref.
Frequently	1.78 (1.35–2.34)	**2.52 (1.47–4.31)**
Perceived religious influence on brain health		
Weak	Ref.	Ref.
Strong	3.58 (2.54–5.05)	1.78 (0.91–3.47)
Frequent engagement in religious activities		
Infrequently	Ref.	Ref.
Frequently	32.82 (19.46–55.36)	**33.42 (18.58–60.11)**

OR, odds ratio; CI, confidence interval.

a Crude OR.

b Adjusted for all variables in the model.

*p < 0.05.*

Upon receiving a brain health test that indicates a risk of developing brain disease, 36% of the participants reported that they would either definitely (15%) or fairly likely (21%) seek religious help. The likelihood of this action was found to be significantly associated with female gender (aOR = 1.64, 95% CI = 1.02–2.63), frequent use of religious activities for brain health (aOR = 2.08, 95% CI = 1.20–3.59), and having a history of COVID-19 (aOR = 1.93, 95% CI = 1.18–3.15); and negatively associated with excellent self-rating of thinking ability (aOR = 0.45, 95% CI = 0.25–0.82) (Table 3).

**Table 3:** Factors associated with the likelihood of seeking religious help upon a brain health test that indicates a risk of developing a brain disease.

Predictors	OR^a^ (95% CI)	OR^b^ (95% CI)
Age		
18–40	Ref.	Ref.
>40	0.61 (0.44–0.85)	0.65 (0.37–1.15)
Gender		
Male	Ref.	Ref.
Female	1.58 (1.16–2.14)	**1.64 (1.02–2.63)**
Marital status		
Unmarried	Ref.	Ref.
Married	0.69 (0.52–0.93)	0.75 (0.46–1.25)
Have chronic illness		
No	Ref.	Ref.
Yes	0.79 (0.54–1.17)	0.84 (0.49–1.43)
Had COVID-19		
No	Ref.	Ref.
Yes	1.64 (1.18–2.28)	**1.93 (1.18–3.15)**
Perceived religious influence on brain health		
Weak	Ref.	Ref.
Strong	2.24 (1.55–3.24)	1.52 (0.91–2.54)
Frequent engagement in religious activities		
Infrequently	Ref.	Ref.
Frequently	2.13 (1.47–3.08)	1.18 (0.68–2.04)
Use of religious activities for brain health		
Infrequently	1	1
Frequently	1.74 (1.31–2.31)	**2.08 (1.20–3.59)**
Self-rated thinking ability		
Not excellent	Ref.	Ref.
Excellent	0.67 (0.5–0.92)	**0.45 (0.25–0.82)**
Self-rated mood balance ability		
Not excellent	Ref.	Ref.
Excellent	0.68 (0.49–0.94)	1.24 (0.66–2.35)

OR, odds ratio; CI, confidence interval.

a Crude OR.

b Adjusted for all variables in the model.

*p < 0.05.*

## Discussion

The results of our study suggest a strong relationship between religion and brain health, and a potential role of religion as a coping strategy and source of support in the face of potential cognitive health challenges. The findings also show that those who frequently engage in religious activities were more likely to believe that religion has a strong influence on brain health. The study demonstrated a notable connection between religion and brain health with more women believing in the influence of religion on cognitive well-being and actively engaging in religious practices for this purpose. This highlights the importance of acknowledging and understanding the role of spirituality in brain health and well-being.

To the best of our knowledge, it is the first study in the UAE or Gulf region to investigate religious beliefs and practices about brain health. Our findings are consistent with the previous reports highlighting the influence of religious beliefs on health-related attitudes and behaviors (Koenig, 2009; Stroope and Baker, 2018; Litalien et al., 2021). Earlier studies investigating the role of religion on brain health or mental health have reported that higher levels of religiousness or spirituality are associated with a significant reduction in anxiety levels, lower depressive symptoms, lower substance use and suicidality, and may also serve to buffer against post-traumatic stress (Litalien et al., 2021; Koenig, 2012). There is also evidence that spiritual health is a foundational component of overall health and well-being, intersecting and integrating with other dimensions of health (Fisher, 2011). A systematic literature review of studies conducted in Canada (Litalien et al., 2021)—a pluralistic and inclusive immigrant society with similarities to the UAE—showed that both institutional and personal religiosity or spirituality were positively associated with mental health. The review also highlighted that health beliefs, behaviors, and decision-making are influenced not only by cultural and religious/spiritual values but also by factors such as age, gender, and geographic location. Additionally, earlier studies have identified a strong protective association between religiosity and subjective well-being, with evidence suggesting that religiosity contributes to mental health resilience among diasporic communities of Muslims, Christians, and Jews in Western societies (Litalien et al., 2021; Ghannam and Gorey, 2023; Rizvi and Hossain, 2017; Snider and McPhedran, 2014). However, a small subset of studies presents mixed or negative associations (Koenig, 2012). Exploring the reasons behind these divergent findings can contribute to our understanding of the underlying mechanisms at play. Through empirical analyses, it has been discovered that spirituality offers additional benefits, such as a heightened sense of purpose and significance in one’s life, which are associated with improved cardiovascular health. These benefits persist even after accounting for other potential psychosocial factors like optimism and positive psychological effects (Kim et al., 2013; Piko and Brassai, 2016).

Our findings show that more women reported the influence of religion on cognitive well-being and actively engaged in religious practices for this purpose. Previous research highlighted the significant gender differences in brain health perceptions, practices, and brain structure and function (Arenaza-Urquijo et al., 2024; Gur and Gur, 2017; Barrientos et al., 2019). Women tend to be more proactive in enhancing brain health, often participating more than men in activities aimed at improving, recovering, and strengthening brain health and resilience. For example, a recent study found that women were three times mor likely to enroll in GBHS (Budin-Ljøsne et al., 2022), and to adopt religious-spiritual and preventative coping strategies compared to men (Mirkovic et al., 2021). Interestingly, earlier studies in the UAE found no significant gender differences in attitudes toward mental health issues, suggesting similar challenges for both genders (Andrade et al., 2022; Ghuloum, 2013). However, cultural and social factors may influence each gender differently. In the UAE, traditional family values and mental health stigma significantly influence these patterns. While marriage and religion are distinct, they frequently intersect, and a relationship between religious participation, marital stability and improved health outcomes has been reported (Waite and Lehrer, 2003). However, the purposeful use of religious activities in married couples specifically for brain health remains understudied. In the UAE’s predominantly Muslim society where religion is embedded in daily life, married individuals may rely more on family and communal religious activities while reducing personal religious observances due to increased social and familial responsibilities (Shukla et al., 2023).

Our results showed that people with COVID-19 were less likely to engage in purposeful use of religious activities for brain health. The most likely reason could be the lockdown and quarantine restrictions that affected the general population, particularly those diagnosed with the virus. During the UAE’s nationwide lockdown, residents were required to stay home except for essential activities. Non-essential businesses, including cafes, gyms, and salons, were closed, and public prayers in mosques, churches, and temples were suspended (Government UAE, 2020). The lockdown and quarantine restrictions disrupted communal worship, leading to decreased participation in religious activities. Qualitative analyses showed that those who decreased their religious devotion during the pandemic often cited inability to worship communally as the primary reason (Leonhardt et al., 2023). Additionally, COVID-19 patients frequently experienced psychological challenges including post-traumatic stress, depression, anxiety, panic, anger, stigma, guilt, and fear of complications or death (Vindegaard and Benros, 2020; AlKuwaiti et al., 2022). While some religious individuals coped better during the pandemic, others reported a decline in religiose engagement (Ayub et al., 2023). These factors together may help to explain our findings of reduced engagement in religious activities for brain health among COVID-19 patients.

We found that half of the study participants reported engaging in religious activities to enhance their brain health. It is worth emphasizing that we did not inquire about the religious beliefs of our participants. Nevertheless, it is essential to recognize that the UAE is primarily a Muslim-majority country, with a significant expatriate population practicing various other faiths, such as Christianity, Buddhism, and Hinduism. Many studies have investigated how these diverse religious backgrounds can impact individuals’ behaviors and beliefs related to health (Koenig, 2012; Piwko, 2021; Villani et al., 2019; Choudry et al., 2018). Being primarily a Muslim country and as most of our respondents were Arabic-speaking, we assume that the majority are Muslims. The Islamic scriptures from the Quran (the Holy book) and the Sunnah (the Prophet Muhammad’s sayings) contain teachings that indicate the importance of certain practices to improve and maintain brain health. Some of these teachings are related to diet and nutrition, mental well-being, physical activity, social cohesion, and hygiene (Assad et al., 2013; Al Khayat, 1997).

The findings from this study have important clinical and public health implications highlighting the proactive approach individuals take to preserve and enhance their cognitive well-being. Furthermore, those who were cognizant of their brain health and were actively engaging in religious activities were more likely to practice religion purposefully for brain health. The relationship between beliefs related to brain health and religiosity, spirituality, and religious practices are intertwined and influential. Although, findings are not entirely consistent across various studies, spirituality and religiosity have been identified as factors that positively influence subjective well-being (Villani et al., 2019; Fredrickson et al., 2008). An increasing body of research provides compelling evidence that individuals who are more deeply engaged in religious practices tend to experience improved physical and mental well-being compared to those who are less involved in religion (Koenig, 2012; Ellison and Levin, 1998; Krause et al., 2011). Furthermore, religious and spiritual beliefs often shape individuals’ understanding and perceptions of brain health. Many religious traditions emphasize the importance of caring for one’s body, including the brain, as a sacred vessel. This perspective is rooted in the belief that the body is a gift from a higher power and should be treated with respect and care (Koenig, 2012). These beliefs may encourage practices such as maintaining a healthy lifestyle, engaging in intellectual pursuits, and seeking medical care when needed.

During periods of stress or illness, including conditions related to the brain, religiosity, spirituality, and engagement in religious practices can function as coping mechanisms. The belief in a higher power, engaging in prayer, meditation, or participating in religious rituals can offer solace, instill hope, and provide emotional support. These factors have the potential to contribute positively to mental well-being and assist individuals in navigating the difficulties associated with brain health challenges (Whitehead and Bergeman, 2020).

Engaging in religious practices frequently involves active participation within a community of fellow believers. This communal experience fosters social support, a sense of belonging, and opportunities for social interaction, all of which can have a positive impact on overall well-being, including brain health (Haim-Litevsky et al., 2023). Previous studies have shown that strong social connections and support systems are associated with improved mental health outcomes and a reduced risk of cognitive decline (Gardener et al., 2021).

Furthermore, people with strong religious beliefs may be more likely to seek spiritual guidance and support in addition to medical interventions when addressing brain health concerns. This integrated approach may lead to a holistic perspective on health and well-being (Bożek et al., 2020). While it is worth noting that the relationship between beliefs related to brain health and religiosity, spirituality, and religious practices can vary across individuals and cultures. However, personal interpretations and experiences within religious and spiritual contexts can also differ significantly (Swihart et al., 2018).

The strengths of our study include a large sample of participants and detailed information about religious beliefs and practices collected through a validated questionnaire. A clear limitation is the cross-sectional nature of the study which does not allow for a causal relationship. Other limitations include recall bias due to self-reported data, and online data collection through convenience sampling strategy which can introduce selection bias. We used convenience sampling approach to address the practical challenges of reaching a geographically diverse population across the UAE to ensure broad representation. Nevertheless, approximately 10% of the participants refused to participate after receiving the survey link, and another 10% decided not to continue after providing consent and answering the first question. Another limitation is the online data collection which may have excluded individuals who lacked access to online surveys or had limited digital access, potentially introducing selection bias. However, in addition to advertising the survey on social media platforms, the research team utilized their personal and professional networks to distribute the survey through invitations and direct messaging to enhance its reach and accessibility. Nevertheless, these limitations in the sampling strategy and data collection process may impact the generalizability of the findings.

## Conclusion

Our study identified factors associated with the purposeful use of religious activities for brain health, suggesting the need for targeted interventions. Individuals more mindful of their brain health and active in religious practices are more likely to use these intentionally for cognitive well-being. Healthcare providers should incorporate spiritually-sensitive assessments into cognitive health screenings, while policymakers could develop frameworks supporting the integration of religious elements into brain health programs. Collaborative initiatives between healthcare institutions and religious organizations, such as joint educational workshops and resource development, should be prioritized to enhance the accessibility and effectiveness of brain health interventions. Future research should explore these relationships through longitudinal studies to understand causal mechanisms linking religious activities and cognitive outcomes. Investigations into neurobiological pathways through which religious practices influence brain function would advance this field. Such efforts can bridge the gap between faith and science, potentially helping to develop more holistic, culturally-responsive approaches to brain health.

## Data Availability

The raw data supporting the conclusions of this article will be made available by the authors, without undue reservation.
